# Strategies to combat cancer drug resistance: focus on copper metabolism and cuproptosis

**DOI:** 10.20517/cdr.2025.41

**Published:** 2025-03-26

**Authors:** Leyi Yao, Baoyi Jiang, Dacai Xu

**Affiliations:** ^1^Zhanjiang Institute of Clinical Medicine, Central People’s Hospital of Zhanjiang, Zhanjiang 524033, Guangdong, China.; ^2^Zhanjiang Central Hospital, Guangdong Medical University, Zhanjiang 524033, Guangdong, China.; ^3^Department of Orthopaedics, The Seventh Affiliated Hospital, Sun Yat-sen University, Shenzhen 518107, Guangdong, China.; ^#^Authors contributed equally.

**Keywords:** Cuproptosis, copper, metabolism, cell death, cancer, drug resistance

## Abstract

Cancer cells often develop tolerance to chemotherapy, targeted therapy, and immunotherapy drugs either before or during treatment. The significant heterogeneity among various tumors poses a critical challenge in modern cancer research, particularly in overcoming drug resistance. Copper, as an essential trace element in the body, participates in various biological processes of diseases, including cancers. The growth of many types of tumor cells exhibits a heightened dependence on copper. Thus, targeting copper metabolism or inducing cuproptosis may be potential ways to overcome cancer drug resistance. Copper chelators have shown potential in overcoming cancer drug resistance by targeting copper-dependent processes in cancer cells. In contrast, copper ionophores, copper-based nanomaterials, and other small molecules have been used to induce copper-dependent cell death (cuproptosis) in cancer cells, including drug-resistant tumor cells. This review summarizes the regulation of copper metabolism and cuproptosis in cancer cells and the role of copper metabolism and cuproptosis in cancer drug resistance, providing ideas for overcoming cancer resistance in the future.

## INTRODUCTION

Cancer cells possess heightened metabolic capacity and energy demand compared to normal cells, continually driving proliferation, metastasis, and drug resistance through metabolic reprogramming^[1-3]^. Tumor drug resistance remains a common challenge in clinical practice, as the inevitable development of drug resistance during chemotherapy, targeted therapy, and immunotherapy significantly complicates cancer treatment. Copper, as one of the vital trace elements for life, maintains enzyme activity by forming copper-containing metalloenzymes or copper-binding proteins, participating in various life processes, including cancer and neurodegenerative diseases^[4]^. As a co-factor for cytochrome c oxidase, copper is indispensable for meeting the energy demands required for rapid cell division, with cancer cells having a greater need for copper. Studies have shown elevated copper concentrations in various cancers, including lung cancer, thyroid cancer, prostate cancer, and breast cancer^[5-7]^.

Growing evidence suggests that copper plays a crucial regulatory role in cell proliferation, autophagy, and cell death in tumors^[8]^. Copper triggers the activation of kinases related to tumorigenesis under moderate concentrations and promotes vessel formation, ultimately promoting tumor development^[9-12]^. As such, copper chelators are studied for their use in cancer treatment^[13]^. Moreover, studies have shown that copper plays a role in promoting cancer drug resistance. For instance, copper transport-related proteins (Ctr1), antioxidant 1 copper chaperone (ATOX1), and copper transporters (ATP7A/B) participate in the translocation of cisplatin, suggesting that targeting the copper transport system may be a strategy to improve treatment efficacies of platinum-containing drugs in cancer chemotherapy^[14-16]^.

Excessive copper intake can trigger the death of tumor cells, a process termed cuproptosis, which is a newly defined form of copper-dependent programmed cell death. Inducing cuproptosis could emerge as a promising strategy for combating cancers^[17]^. Currently, it has been discovered that copper ionophores, copper-based nanomaterials, and others can enhance drug sensitivity in drug-resistant tumor cells by triggering cuproptosis or directly killing drug-resistant tumor cells^[18]^. This review elucidates copper metabolism regulation and discusses the possibility of overcoming tumor resistance by targeting copper metabolism and promoting cuproptosis.

## COPPER METABOLISM IN CANCER

**Figure 1:**
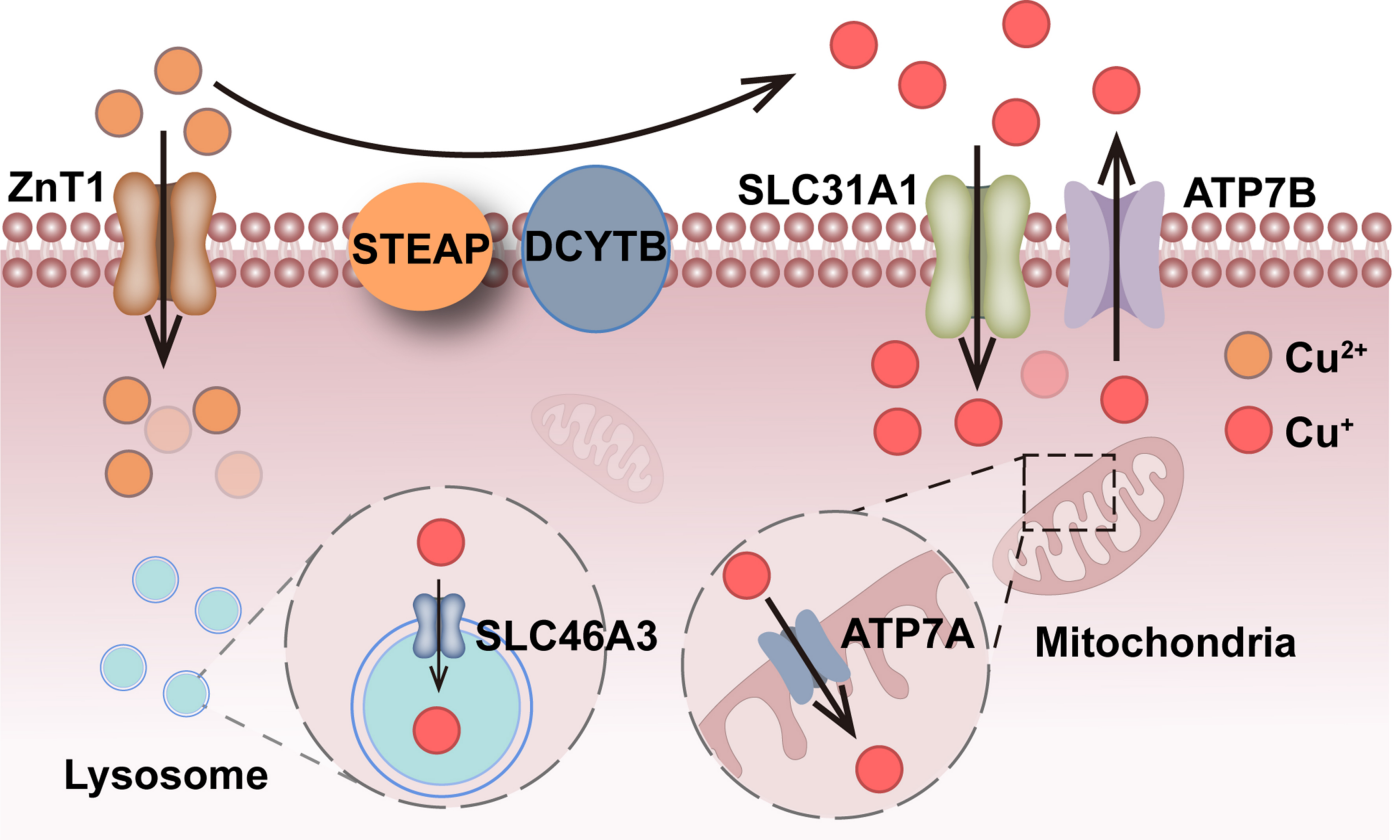
Copper redox, oxidation, and absorbance in cells. ZnT1 directly transports extracellular Cu^2+^ into cells. Extracellular Cu^2+^ could be reduced to Cu^+^ via STEAP and then transported into cells via SLC31A1, while ATP7B opposes this process via excreting Cu^+^. Intracellular Cu^+^ is absorbed into lysosomes and mitochondria via SLC46A3 and ATP7A, respectively. ZnT1: Solute carrier family 30 member 1/Slc30a1, a zinc and copper transporter; STEAP: 6-transmembrane epithelial antigen of prostate, comprises STEAP1, STEAP2, STEAP3, and STEAP4; Dcytb: duodenal cytochrome b; SLC31A1: solute carrier family 31 member 1, Ctr1: copper transport protein 1; ATP7B: ATPase copper-transporting beta; SLC46A3: solute carrier family 46 member 3; ATP7A: ATPase copper-transporting alpha.

Copper exists in two forms in the human body: copper ion (Cu^+^, reduced state) and copper ion (Cu^2+^, oxidized state) [Figure 1]. The steady-state balance of the two forms of copper is important for exerting its effect. The metabolic progress of copper is related to the inter-conversion between Cu^+^ and Cu^2+^, which is achieved by providing or accepting a single electron^[19]^.

The solute carrier family 30 member 1 (SLC30A1/ZnT1) is a plasma membrane transporter that exports zinc (Zn^2+^) from cells while also regulating Cu^2+^ uptake and is necessary for cuproptosis^[20]^. Moreover, the human 6-transmembrane epithelial antigen of the prostate (STEAP) family, ferredoxin 1 (FDX1), and the histone H3-H4 tetramer reduced Cu^2+^ to Cu^+^, while the multicopper oxidase CueO oxidized Cu^+^ to Cu^2+^^[8,21-23]^. Recently, solute carrier family 46 member 3 (SLC46A3) has been identified as a lysosomal protein whose expression is induced by 2,3,7,8-tetrachlorodibenzo-p-dioxin (TCDD), resulting in reduced cytosolic copper levels in the liver of mice^[24]^.

Maintaining copper homeostasis in the body is crucial for health. Copper is absorbed from food in the small intestine, passed through the portal vein, and transported via plasma proteins such as albumin and ceruloplasmin to the liver, where it is bound to metallothioneins (MTs) and glutathione (GSH), and stored in hepatocytes. In the small intestine, Cu^2+^ is reduced to Cu^+^ by metal reductases such as STEAP and duodenal cytochrome b (Dcytb) [Figure 1]. It is then transported into intestinal cells by copper transport protein 1 [Ctr1, also known as solute carrier family 31 member 1 (SLC31A1)]. The human gene copper transport protein 2 [Ctr2, also known as solute carrier family 31 member 2 (SLC31A2)], which is highly homologous to the *Ctr1* gene, acts as a copper transporter under certain conditions, maintaining intracellular copper homeostasis^[25]^. Multi-database analysis suggests that Ctr1 may be a tumor predictive biomarker^[26]^. Beyond its role in copper transport, Ctr1 also serves as an important transporter for cisplatin, a cancer chemotherapy drug, to enter yeast and mammalian cells^[27,28]^. The response rate of platinum therapy has been shown to be associated with high levels of Ctr1 expression. Therefore, lower levels of Ctr1 have also been observed to be related to increased cisplatin resistance in tumors, whereas higher Ctr1 expression is linked to a greater sensitivity to cisplatin treatment^[29,30]^. In chemotherapy-resistant patients, an elevated Ctr2/Ctr1 ratio portends a worse prognosis^[31]^. Thus, investigating key regulatory factors in copper metabolism offers great potential for driving advancements in cancer research.

**Figure 2:**
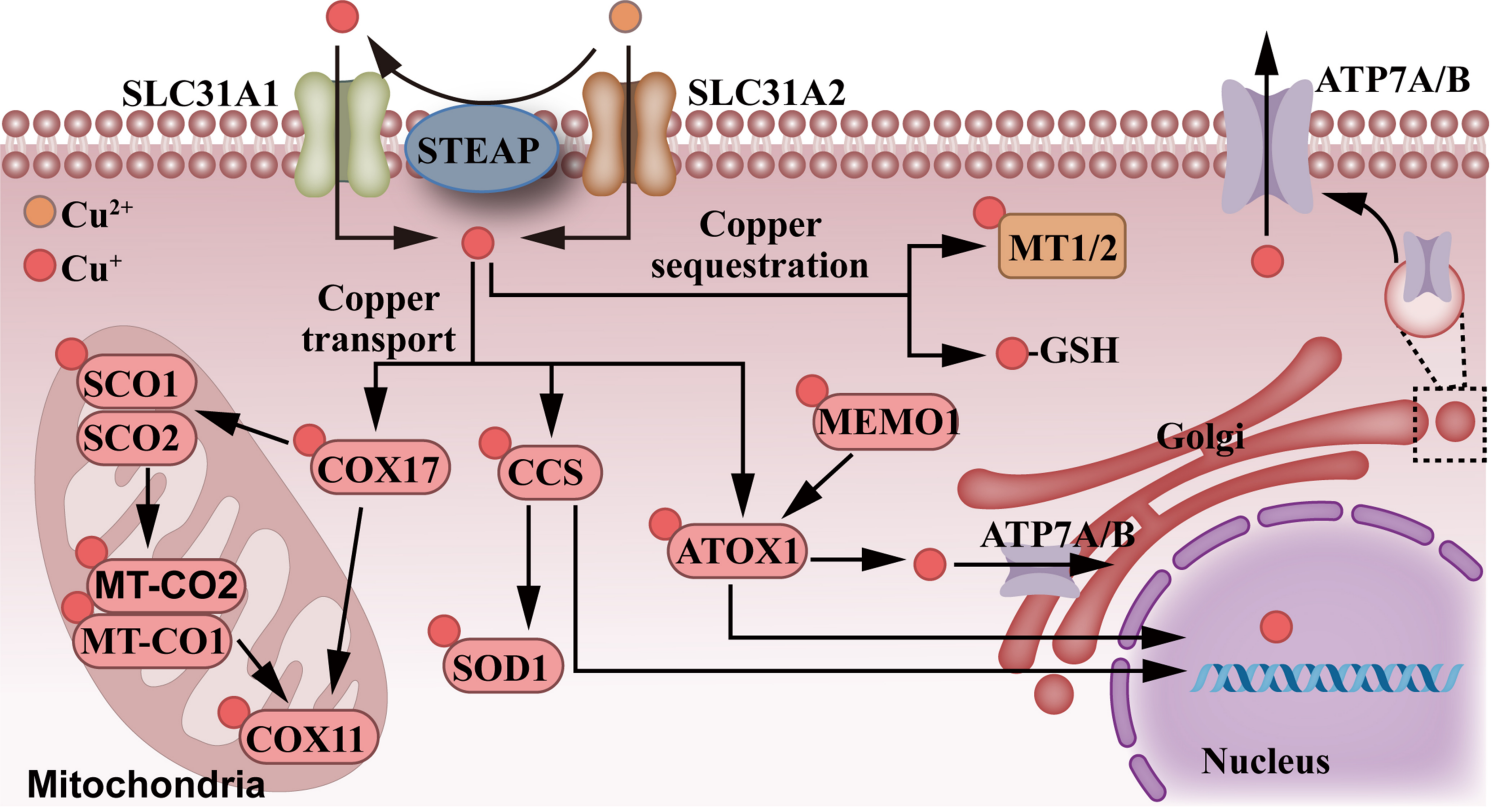
The signaling pathways of copper metabolism. Extracellular Cu^2+^ is reduced to Cu^+^ via STEAP. Both Cu^2+^ and Cu^+^ are transported into cells via corresponding transporters. Intracellular copper is sequestrated via MT1/2 or GSH or transported via COX17, CCS, SOD1, ATOX1, MEMO1, and ATP7B. Mitochondrial copper functions via SCO1, SCO2, MT-CO1, MT-CO2, and COX11, while nuclear copper can regulate gene expression. STEAP: 6-Transmembrane epithelial antigen of prostate, comprises STEAP1, STEAP2, STEAP3 and STEAP4; MT1/2: metallothionein 1/2; GSH: glutathione; COX17: cytochrome c oxidase copper chaperone COX17; CCS: copper chaperone for superoxide dismutase; SOD1: superoxide dismutase 1; ATOX1: antioxidant 1 copper chaperone; MEMO1: mediator of cell motility 1; ATP7B: ATPase copper-transporting beta; SCO1: synthesis of cytochrome C oxidase 1; SCO2: synthesis of cytochrome C oxidase 1; MT-CO1: mitochondrially encoded cytochrome c oxidase I; MT-CO2: mitochondrially encoded cytochrome c oxidase II; COX11: cytochrome c oxidase copper chaperone COX11.

Copper metabolism is meticulously controlled by multiple chaperones, including ATOX1, cytochrome c oxidase copper chaperone (COX17), and copper chaperone for superoxide dismutase (CCS), which transport copper to the Golgi apparatus, mitochondria, and copper/zinc superoxide dismutase enzymes (SOD), respectively^[32]^ [Figure 2]. CCS is a copper-binding protein that interacts with copper-zinc superoxide dismutase 1 (SOD1), located in the cytoplasm and the intermembrane space of mitochondria. By forming a heterodimer with SOD1, it transfers copper from the cytoplasm to SOD1, activating SOD1 and eliminating reactive oxygen species (ROS) in the organism^[33,34]^. In addition, CCS also delivers copper ions to the cell nucleus^[35]^. Research has reported that CCS promotes the growth and migration of breast cancer cells by regulating ROS-mediated ERK1/2 activity^[36]^. Inhibiting CCS would block the growth of lung cancer and leukemia cells^[37]^. Although the potential mechanisms by which CCS is linked to tumorigenesis remain to be explored, existing evidence points to CCS as a potential target for tumor therapy.

COX17 assembles cytochrome c oxidase in mitochondria as a copper chaperone. The transportation of Cu^+^ into mitochondria mainly depends on COX17. COX17 transfers Cu^+^ from the cytoplasm to the mitochondrial membrane proteins: synthesizing cytochrome c oxidase 1 (SCO1) and 2 (SCO2), thereby inserting copper into the MT-CO2/COX2 encoded by the mitochondria^[38]^. According to reports, COX17 can inhibit the progression of renal fibrosis and exacerbate Alzheimer’s disease by regulating copper levels^[39,40]^. In addition, it has been suggested that COX17 may serve as a therapeutic target for non-small cell lung cancer^[41]^.

ATOX1 is an important copper chaperone protein, antioxidant, and transcription factor whose oxidative function and transcriptional activity both depend on its capacity to bind copper. ATOX1 regulates copper distribution within the cytoplasm; it can acquire copper ions from Ctr1, CCS, glutathione 1 (GRX1), and GSH, transporting them to the Golgi apparatus or nucleus. The copper-binding capacity and transport activity of ATOX1 are regulated by GSH. ATOX1 has been shown to directly interact with ATPases (ATP7A/B) in a copper-dependent manner. When cellular copper overload occurs, ATOX1 transports copper via ATP7A/B transporters to the Golgi apparatus or lysosomes, thereby facilitating the export of excess copper^[42-44]^. Studies show that ATOX1 promotes cell proliferation, migration, and autophagy. When activated by copper, ATOX1 undergoes nuclear translocation, DNA binding, and activation, thereby promoting cell proliferation^[45]^. In addition, ATOX1 binds to cisplatin through direct interaction, leading to the vesicular sequestration of cisplatin, which may cause drug resistance in tumor cells^[46,47]^. Blockage of the copper transportation functions of ATOX1 and CCS results in copper homeostasis imbalance, significantly inhibiting cancer cell proliferation and tumor growth, and increasing the sensitivity of cancer cells to therapeutic drugs^[37,48,49]^. Given the high dependence of cancer cells on copper, establishing copper chaperone molecules as new therapeutic targets for cancer treatment is of great significance.

Recently, SLC46A3 has been found to be localized in the lysosomes and is constitutively expressed in the liver of wild-type mice. According to copper analysis in the liver and cell lines, SLC46A3 may transport copper into the lysosomes and regulate intracellular copper levels^[24]^. When cellular copper overload occurs, copper is transported by the copper-transporting ATPases (ATP7A and ATP7B) located in the Golgi apparatus, loading copper into the Golgi vesicles. Finally, the vesicles fuse with the plasma membrane and release copper into the extracellular space. Research has shown that ATP7A and ATP7B are associated with drug resistance in various tumors^[32,50]^, providing potential targets for overcoming tumor resistance challenges in future clinical practice.

## CUPROPTOSIS IN CANCER

**Figure 3:**
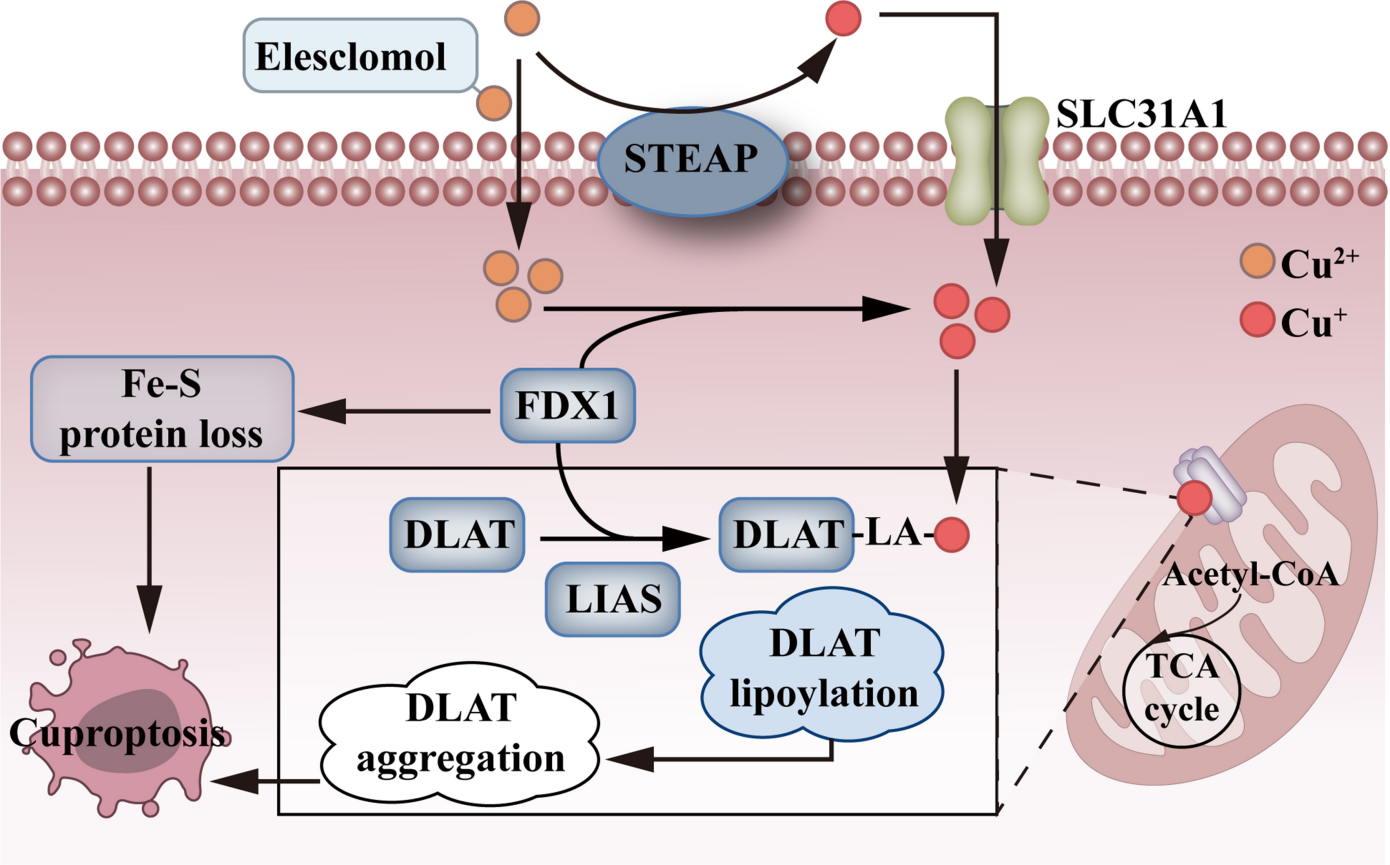
The mechanism of cuproptosis. Copper ionophore elesclomol directly transports Cu^2+^ into cells and then Cu^2+^ is reduced to Cu^+^ via FDX1. After the reduction of Cu^2+^ into Cu^+^ via STEAP, Cu^+^ is pumped into cells via SLC31A1. FDX1 and LIAS induce DLAT lipoylation and aggregation, inducing the damage of the TCA cycle. As such, cells are dead via cuproptosis. FDX1: Ferredoxin 1; STEAP: 6-transmembrane epithelial antigen of prostate, comprises STEAP1, STEAP2, STEAP3, and STEAP4; SLC31A1: solute carrier family 31 member 1; LIAS: lipoic acid synthetase; DLAT: dihydrolipoamide S-acetyltransferase; TCA: tricarboxylic acid; LA: lipoic acid.

Copper is a crucial component in the formation and maintenance of various copper enzymes, playing a pivotal role in cancer cell metabolism, which sustains their rapid growth. As a result, cancer cells exhibit an increased dependency on copper. However, excessive copper can induce tumor cell death, which is called cuproptosis [Figure 3]. Excessive intracellular Cu^2+^ can be transported into mitochondria, where FDX1 reduces Cu^2+^ to Cu^+^. Subsequently, Cu^+^ directly interacts with the lipidated components of the tricarboxylic acid (TCA) cycle, leading to increased fatty acylation and aggregation of the mitochondrial protein DLAT (dihydroceramide S-succinyltransferase). This leads to a decrease in the stability of Fe-S cluster proteins, resulting in proteotoxic stress and, ultimately, cell death^[17]^. Copper also induces ferroptosis by promoting the generation of ROS and facilitating the degradation of glutathione peroxidase 4 (GPX4) in cancer cells^[51]^.

### Copper ionophores for inducing cuproptosis in cancer cells

Currently, elesclomol and disulfiram are two common copper ion carriers. Elesclomol, originally developed by Synta Pharmaceuticals as a chemotherapeutic adjuvant, was long believed to function by generating ROS and inducing Hsp70^[52]^. However, it was later found to bind Cu^2+^ and facilitate its intracellular delivery. The specific mechanism by which copper overload induces cell death remained unclear until the discovery of cuproptosis. The target of elesclomol, ferredoxin-1 (FDX1), subsequently reduces Cu^2+^ to its Cu^+^ form. This reduction leads to the accumulation of copper-bound lipoylated mitochondrial proteins and the destabilization of iron-sulfur cluster proteins, resulting in proteotoxic stress and, ultimately, cell death^[53]^. Thus, elesclomol primarily targets mitochondrial metabolism, and cells with high mitochondrial metabolism are very sensitive to elesclomol^[54,55]^. Harnessing the metabolic plasticity of cancer cells, inhibiting glycolysis, and enhancing reliance on mitochondrial metabolism can ultimately increase the effectiveness of elesclomol in suppressing tumors^[56,57]^. Clinical trials have already validated the safety of elesclomol and its selective cytotoxicity toward cancer cells^[58]^.

Disulfiram is an aldehyde dehydrogenase (ALDH) inhibitor that was first approved for the treatment of alcoholism in 1951, and it is currently gaining attention in the field of cancer research^[59,60]^. Disulfiram can form with copper as disulfiram-Cu complexes that induce cancer cell death and inhibit cancer cell migration, invasion, and angiogenesis^[8,61,62]^. Additionally, disulfiram-Cu can simultaneously induce cuproptosis and ferroptosis in hepatocellular carcinoma cells^[63]^. The combined use of disulfiram and copper has a stronger inhibitory effect on tumor growth than the use of disulfiram or copper alone^[64]^. Other copper ionophores include 7-iodo-5-chloro-8-hydroxyquinoline (CQ) and bidentate thiosemicarbazones. CQ-Cu exhibits great selectivity against prostate cancer cells, exerting its anticancer effects by inducing X-linked inhibitor of apoptosis (XIAP) degradation in these cancer cells^[65]^. The dithiocarbamates (SMTMs) ligands, diacetylbis [N4-methylthiosemicarbazonato] Cu(II) (Cu-ATSM) and glyoxalbis [N4-methylthiosemicarbazonato] Cu(II) (Cu-GTSM), have been investigated as potential anticancer drugs for prostate cancer cells both *in vitro* and *in vivo*^[66]^.

### Nanoparticles for inducing cuproptosis in cancer cells

Owing to the unique dimensions and performance advantages of nanomaterials, researchers employed nanocarriers as the foundation for a delivery system to precisely deliver copper, copper ionophores, and other anticancer drugs to cancer cells. This aims to explore tumor treatment strategies based on nanomedicines-induced cuproptosis^[18]^. For instance, elesclomol-loaded copper oxide (CuO) nanoparticles (NPs) induce cuproptosis, enhance tumor immune response, and exhibit anti-mouse melanoma effects^[67]^. TP-M-Cu-MOF/siATP7A can effectively silence the *ATP7A* gene, increasing copper intake, thereby inducing cuproptosis and enhancing antitumor efficacy^[68]^. The polydopamine nanomaterials loaded with high-concentration copper ions (PDA-DTC/Cu) can enhance copper uptake, inhibit the expression of ATP7A and ATP7B, and improve tumor immune therapy^[69]^. Cu-doped polypyrrole (CuP) nanospheres containing bis-2-(5-phenylacetamido-1,2,4-thiadiazol-2-yl)ethyl sulfide (BPTES) can enhance cuproptosis and immunotherapy, leading to inhibiting both primary and metastatic tumors^[70]^. Glucose oxidase copper-based nanomaterials GOx@[Cu(tz)], through their diverse cooperative effects, in turn, treat bladder cancer^[71]^. A transparent hydrogel-modified metal-organic framework loaded with doxorubicin (DOX) and calcium peroxide has been developed to form a self-enhancing bimetallic Mito-Jammer (also known as HA‐CD@MOF NPs). This enhances ROS storms and mitochondrial damage, thereby sensitizing cancer cells to cuproptosis, activating robust immunogenic cell death, and suppressing tumor metastasis^[72]^. Nano-sized coordination polymer particles Cu/TI target mitochondria, effectively inducing copper death and PD-L1 downregulation and inhibiting the growth of colorectal cancer (CRC) and triple-negative breast cancer^[73]^. Nanomaterials can induce different types of cell death simultaneously to exert anticancer effects. CuO2@G5-BS/TF modulates the tumor microenvironment to achieve targeted TNBC magnetic resonance imaging, enhancing ferroptosis, cuproptosis, and chemodynamic therapy^[74]^. Copper-based nanomaterials have emerged as a promising strategy in cancer therapy as they induce cuproptosis and enhance immunotherapy efficacy. By delivering copper ions directly to tumor cells, these nanomaterials can simultaneously modulate the tumor microenvironment and stimulate the immune system, thereby amplifying anticancer effects. Collectively, copper-based nanomaterials play a significant role in improving the efficiency of cancer treatment [Table 1].

**Table 1:** Cuproptosis-related NPs for cancer treatment

**NPs**	**Tumor types**	**effects**	**Ref.**
ES@CuO	Melanoma	Induce cuproptosis, enhance immune response	[67]
TP-M-Cu-MOF/si ATP7A	Small cell lung cancer	Induce cuproptosis, improved therapeutic efficacy in SCLC brain metastasis	[68]
PDA-DTC/Cu	Breast cancer	Enhance cuproptosis, stimulate the immune response, relieve the tumor immunosuppressive microenvironment	[69]
Cu-doped polypyrrole nanoparticles (CuP) nanosystem (PCB)	Breast cancer	Induce cuproptosis, improve immunogenic cell death, suppress both primary and distant tumors	[70]
GOx@[Cu(tz)]	Bladder cancer	Induce cuproptosis, develop photodynamic synergistic therapy	[71]
HA‐CD@MOF NPs	Breast cancer	Increase cuproptosis sensitization, induce immunogenic cell death, suppress tumor metastasis	[72]
Cu/TI	Colorectal carcinoma, triple‐negative breast cancer	Induce cuproptosis, induce immunogenic cancer cell death and reduce PD‐L1 expression	[73]
CuO_2_@G5-BS/TF	Triple‐negative breast cancer	Induce ferroptosis, cuproptosis, and enhance chemodynamic therapy	[74]
CS/E-C@DOX Nanoplatform	breast cancer	Induce cuproptosis, inhibit the stemness of cancer cells	[75]
CuET NPs	Non-small cell lung cancer	Induce cuproptosis	[76]

NPs: Nanoparticles; MOF: metal-organic framework; ATP7A: ATPase copper-transporting alpha; SCLC: small cell lung cancer; PDA-DTC: polydopamine- diethyldithiocarbamate; PD-L1: CD274 molecule; CS: chondroitin sulfate; DOX: doxorubicin.

### Other agents for inducing cuproptosis in cancer cells

Beyond classical copper ionophores, an increasing number of chemical agents have been found to exert anticancer effects by regulating copper metabolism and inducing cuproptosis. Therefore, the identification of novel cuproptosis-regulated chemicals may provide new strategies for cancer treatments.

LGOd1 interferes with cellular copper homeostasis by disrupting the copper chaperone protein CCS, thereby inducing cuproptosis in liver cancer cells^[77]^. Sorafenib, the first-line treatment drug for liver cancer, and erastin can promote cuproptosis in liver cancer by inhibiting FDX1 and upregulating the lipoic acid modification of proteins^[78]^. Eupalinolide B (EB) from Eupatorium lindleyanum enhances the inhibitory effect of elesclomol on pancreatic cancer^[79]^. Zinc pyrithione can induce the death of TNBC cells by disrupting the homeostasis of copper metabolism and triggering DLAT aggregation^[80]^. In addition, the protein synthesis inhibitor anisomycin, which binds to the 60S ribosomal subunit, has been found to lead to the inactivation of the transcriptional activity of FDX1, DLD, DLAT, and PDHB, potentially contributing to cuproptosis in ovarian cancer stem cells^[81]^. Recent studies have also found that curcumin, a natural compound extracted from turmeric, can act as a copper ionophore, promoting copper-induced cell death in CRC cells^[82,83]^. In addition, curcumin exerts anti-liver cancer effects through ferroptosis and cuproptosis^[84]^. These findings highlight the therapeutic potential of targeting copper-dependent cell death pathways, such as cuproptosis, in various cancers.

## ROLE OF COPPER METABOLISM AND CUPROPTOSIS IN TUMOR DRUG RESISTANCE

### Copper chelators overcome cancer drug resistance

Copper level increases in cancers and is associated with tumor grade^[85]^. Moreover, copper metabolism is correlated with drug resistance in tumors^[27,86-90]^. For example, loss of Ctr1 reduces the absorption of cisplatin into cells^[89]^, and low Ctr1 levels are associated with poor clinical response to cisplatin^[91]^. Similarly, high ATP7A expression is linked to reduced chemotherapy sensitivity in colon cancer, as it can directly efflux DOX and SN-38 from cells^[90]^. Therefore, copper chelators act as promising agents for overcoming anticancer drug resistance. Indeed, copper chelator tetrathiomolybdate boosts the antitumor effect of cisplatin via fostering the uptake of cisplatin in cervical cancer and enhances the anticancer effect of metformin in breast cancer^[91,92]^. Another copper chelator, curcumin, also promotes the susceptibility of cisplatin in lung cancer cells^[93]^. Moreover, copper chelator JYFY-001 augments the antitumor effect of PD-1 inhibitors in CRC^[94]^. Thus, copper chelators enhance drug sensitivity by disrupting copper metabolism and facilitating drug uptake.

### Copper ionophores combat cancer drug resistance

**Figure 4:**
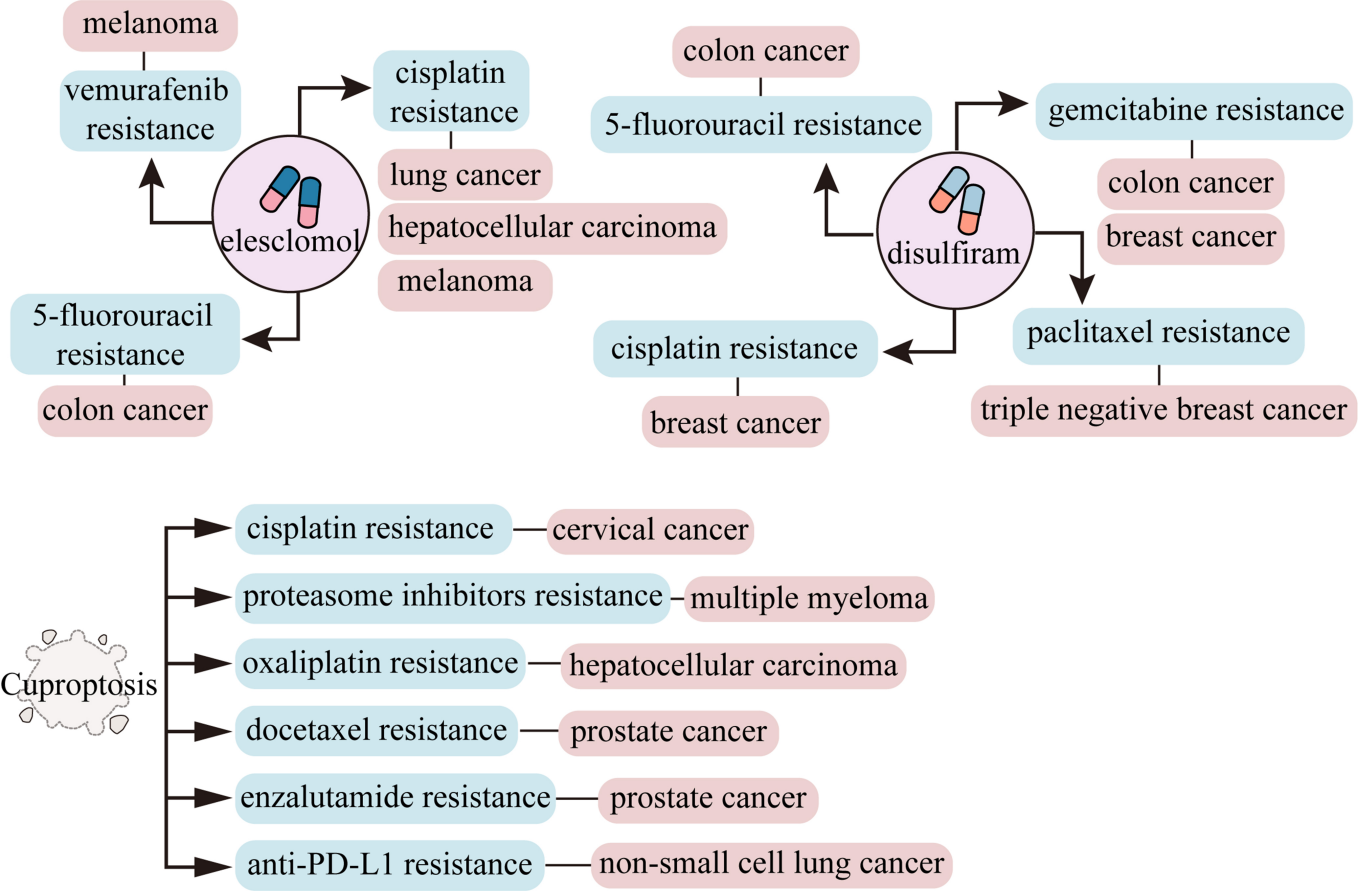
Copper ionophores and cuproptosis combat tumor drug resistance. Copper ionophore elesclomol overcomes cisplatin, 5-fluorouracil and vemurafenib resistance in the indicated cancers. Copper ionophore disulfiram bypasses cisplatin, 5-fluorouracil, paclitaxel and gemcitabine resistance in the indicated tumors. Cuproptosis inducers combat cisplatin, proteasome inhibitors, oxaliplatin, docetaxel, enzalutamide and anti-PD-L1 resistance in the indicated cancers. PD-L1: CD274 molecule.

The induction of cuproptosis may be an effective strategy to overcome drug resistance in cancers [Figure 4]. Indeed, studies demonstrated that copper ionophore elesclomol can trigger cisplatin-resistant cell death in lung cancer, melanoma, and hepatocellular carcinoma^[95-97]^. In addition, 5-fluorouracil-resistant colon cancer cells and vemurafenib-resistant melanoma cells were also highly sensitive to elesclomol^[98,99]^. Under the dual pressure of chemotherapeutic drugs and elesclomol, it helps to prevent cancer cells from developing drug resistance. However, a Phase II clinical trial investigating the combination of elesclomol and paclitaxel for treating platinum-resistant ovarian, fallopian tube, or primary peritoneal cancer showed no significant benefit, as only a small proportion of patients responded^[100]^. Another clinical trial on paclitaxel alone or in combination with elesclomol in advanced melanoma demonstrates that elesclomol does not prolong the progression-free survival induced by paclitaxel^[101]^. Paclitaxel alone or in combination with elesclomol shows similar toxicity in refractory solid tumors in a phase I clinical trial^[102]^. These clinical trials contradict the effects observed *in vitro*, which may result from individual differences, differences in dosage, and the complex drug metabolism mechanisms *in vivo*. Therefore, chemotherapy combined with elesclomol can overcome the issue of tumor drug resistance *in vitro*.

Given that elesclomol provokes cell death in drug-resistant cancers, other copper ionophores may also function in cancer drug resistance. For instance, disulfiram overcomes paclitaxel resistance in triple-negative breast cancer cells^[103]^. Moreover, disulfiram fuels the tumor-killing action of cisplatin by retarding the tumor stemness and reshaping the vulnerability of ALDH^+^ stem-like cells to cisplatin in breast cancer^[104]^. Furthermore, the disulfiram-Cu complex boosts the sensitivity of gemcitabine-resistant cell lines^[105]^, and disulfiram-mediated blockage of NF-κB activity enhances the sensitivity of 5-fluorouracil in human CRC cell lines^[106]^. Although disulfiram shows a similar antitumor effect as elesclomol, its clinical trial performance is not good.

### Cuproptosis enhances drug sensitivity in drug-resistant cancer cells

Copper ionophores also foster susceptibility of drug-resistant cancer cells by inducing cuproptosis [Figure 4]^[107]^. For example, elesclomol-Cu inhibits autophagy and promotes cell retention in the G2/M phase, thereby reinforcing the chemosensitivity of docetaxel in prostate cancer^[108]^. Copper ionophore significantly boosts the cytotoxicity of enzalutamide in enzalutamide-resistant cells, providing an effective option for the treatment of castration-resistant prostate cancer (CRPC) cells, especially enzalutamide-resistant CRPC^[109]^. Disulfiram combined with anti-PD-L1 circumvents NSCLC anti-PD-L1 resistance by regulating the HIF-1 signaling pathway through ATP7B^[110]^. Other chemicals also attenuate the drug resistance in cancer. As a recent study reported, baicalein exacerbates cuproptosis via AKT pathway blockage, and then circumvents the cisplatin resistance in cervical cancer cells^[111]^. Copper and iron homeostasis influences each other, leading to an interplay between cuproptosis and ferroptosis^[4]^. For example, copper also triggers ferroptosis by boosting the product of ROS and enhancing the autophagy-associated degradation of GPX4 in cancer cells^[51]^. The combination of cuproptosis inducer elesclomol-Cu and ferroptosis inducer imidazole ketone erastin (IKE) facilitates both cuproptosis and ferroptosis in myelodysplastic syndromes^[112]^. Additionally, the ferroptosis inducers erastin and sorafenib promote the cuproptosis effects of copper ionophores in liver cancer^[78]^. Therefore, these findings suggest that cuproptosis inducers and ferroptosis inducers provide a novel strategy for overcoming drug resistance.

Non-coding RNAs play a critical role in regulating cellular responses, influencing both oncogenesis and drug resistance. Among them, HOX antisense intergenic RNA (HOTAIR) is one of the most well-studied oncogenic long non-coding RNAs (lncRNAs), implicated in cisplatin resistance in lung adenocarcinoma as well as trastuzumab and DOX resistance in breast cancer^[113,114]^. Additionally, lncRNA H19 has been identified as a driver of tamoxifen resistance in breast cancer^[115]^. Recently, cuproptosis-related lncRNAs have been identified and integrated into prognostic models. For instance, a model featuring six cuproptosis-related lncRNAs: AC026412.3, AC125437.1, AL353572.4, MKLN1-AS, TMCC1-AS1, and SLC6A1-AS1 predicts both the prognosis and immunotherapy outcomes in hepatocellular carcinoma patients^[116]^. Additionally, non-coding RNAs/DNAs also modulated the drug resistance via cuproptosis in cancers [Figure 4]. For example, MUC20 triggers cuproptosis and reverses proteasome inhibitor resistance by thwarting the expression of cuproptosis-negative regulator CDKN2A, blocking IGF-1R lactation and abrogating MET activation in multiple myeloma cells^[117]^. LINC02362/ hsa-mir-18a-5p/FDX1 axis provokes cuproptosis and enhances the sensitivity of oxaliplatin in hepatocellular carcinoma, providing a promising therapeutic approach to bypass oxaliplatin resistance in hepatocellular carcinoma^[118]^. These non-coding RNAs/DNAs may be targets for bypassing drug resistance in cancers. Since non-coding RNAs perform a variety of roles, their silencing (in the case of oncogenes) or overexpression (for tumor suppressors) has emerged as an attractive study topic in recent years. For instance, the silencing of HOTAIR by siRNA sensitizes breast cancer to trastuzumab and DOX^[119,120]^. Focusing on the regulatory non-coding RNAs related to cuproptosis may provide new approaches to overcome tumor drug resistance.

Studies show that nanomaterials overcome tumor resistance by promoting cuproptosis. For example, ellagic acid (EA), Cu^2+^, DOX, and chondroitin sulfate (CS) form CS/E-C@DOX Nanoplatform (CS/NPs). CS/NPs show excellent antitumor effects by inducing cuproptosis and significantly inhibiting the stemness of cancer cells without body weight loss, suggesting strong potential to confer cancer chemoresistance^[75]^. CuET NPs bypass cisplatin resistance in A549 cells by driving cuproptosis without body weight loss^[76]^. The nanomaterials show low toxicity but high efficiency in overcoming drug resistance. Targeting cuproptosis may be a novel antitumor treatment and therapeutic strategy to circumvent drug resistance in cancers [Table 2].

**Table 2:** Cuproptosis-related compounds for overcoming cancer drug resistance

**Compounds**	**Tumor types**	**Enhance drug efficacy**	**Ref.**
Tetrathiomolybdate	Cervical cancer, breast cancer	Cisplatin, metformin	[91,92]
Curcumin	Lung cancer	Cisplatin	[93]
JYFY-001	CRC	PD-1 inhibitor	[94]
Elesclomol	Lung cancer, melanoma, hepatocellular carcinoma, colon cancer, CRPC	Cisplatin, 5-fluorouracil, vemurafenib, docetaxel, enzalutamide	[95-99,108,109]
Disulfiram	Triple-negative breast cancer, breast cancer, colon cancer, CRC, CRPC, non-small cell lung cancer	Paclitaxel, cisplatin, 5-fluorouracil, enzalutamide, gemcitabine	[103-106,109,110]
Baicalein	Cervical cancer	Cisplatin	[111]
CS/E-C@DOX nanoplatform	Breast cancer	Adriamycin	[75]
CuET NPs	Non-small cell lung cancer	Cisplatin	[76]

CRC: Colorectal cancer; CRPC: castration-resistant prostate cancer; CS: chondroitin sulfate; DOX: doxorubicin; NPs: nanoparticles.

## CONCLUSION AND PROSPECT

Copper is an essential element in the human body that participates in a variety of vital processes. Existing in both oxidized and reduced states, copper exerts dual effects *in vivo.* It can inhibit multiple cancer-promoting pathways while also facilitating tumor development under certain conditions. The imbalance of copper homeostasis can cause a variety of diseases, including cancers. At present, a series of copper ionophores as anticancer drugs have attracted the close attention of researchers. Although the antitumor effect of copper ionophores is exciting, there are still challenges in their clinical translation.

Copper and copper-based nanomaterials have been widely used in nanomedicine. Copper-based nanomaterials prepared by new nanotechnology have excellent physical and chemical properties, and can be used in various biomedical applications. The method of inducing cuproptosis combined with immunotherapy shows good results in tumor treatment. The biodegradation rate of copper-based nanomaterials is low, and long-term existence in the body may produce potential toxicity and adverse effects^[121,122]^, which has become a major challenge for tumor treatment. Further optimizing the biocompatibility of copper-based nanomaterials, improving the biodegradability, and realizing the design of multifunctional nanoplatforms are the research directions for exploring antitumor therapy in the future.

Copper ionophores and copper-based nanomaterials, alone or in combination with other antitumor methods, can treat cancer, especially to overcome cancer drug resistance. Small molecule compounds and NPs kill cancer cells by inducing cuproptosis, which will provide new ideas for the development of anticancer drugs by driving cuproptosis in the future^[107]^. However, we also face the potential issue of drug resistance. To that end, further research is needed to elucidate the physiological roles and mechanisms of copper ions in human beings, investigate the molecular mechanisms of cuproptosis, and identify new cuproptosis inducers. These efforts could provide new research directions for overcoming tumor drug resistance.

## DECLARATIONS

### Copyright

© The Author(s) 2025.
